# Supplementary material for: sMAdCAM: IL-6 Ratio Influences Disease Progression and Anti-Viral Responses in SARS-CoV-2 Infection
Source: Front Immunol. 2021 Jun 14;12:619906. doi: 10.3389/fimmu.2021.619906 (PMC8236632; doi:10.3389/fimmu.2021.619906)

Index

1. Supplemental Table 1. Study cohort characteristics
2. Supplemental Figure 1: Distribution of plasma TNF-α across various serological groups of SARS-CoV-2 infected individuals.
3. Supplemental Figure 2: Association of IL-6 with other cytokines and Ct for RT-PCR of different SARS-CoV-2 gene.
4. Supplemental Figure 3: Association of TNF-α with other cytokines and Ct for RT-PCR of different SARS-CoV-2 gene.
5. Supplemental Figure 4: Analysis of circulating lipopolysaccharides (LPS) with SARS-CoV- 2 disease progression

Supplemental Table 1. Study cohort characteristics

Sample Id	Age (Yrs)	Gender (M/F)	Ct Value			Days post PCR test until sampling	(IgM)	(IgG)	CLIA IgG (Value)	Co-morbidities	Medication
			N	O	S/R						
U1	51	M	32	32	33	3	+	+	3.16	HIV+	Cefixime, Pan 40, Pcm, MVBC, Vit C (500mg), cough syrup
U2	55	M	19	21	20	9	-	-	1.55	None	PCM 500, Pan 40, Levocet 5MG
U3	34	M	25	26	25	0	-	-	-	TRF not traceable	NA
[a]U4	38	F	31	29	28	11	-	+	5.94	Diabetes mellitus	DD, MUBC, Metformin 500mg, Insulin , Pantop 40mg
U5	38	M	33	32	34	2	+	+	4.72	Diabetes	Azithromycin,HCQ, Tamiflu, Vit C, Augmentin,
U6	72	F	28	25	26	11	+	+	5.28	Diabetes, Hypertension	Azec, Pan40, Glimi, MVBC, Amlodipin, Inj PJ
U7	35	F	33	32	33	3	+	+	6.9	None	NA
U8	40	F	-	-	-	17	+	+	2.6	None	Pan , MVBC
[a]U9	70	M	34	32	32	5	-	-	7.19	None	Not available
U10	63	M	25	26	25	2	-	-	1.57	Diabetes	HCQ, Tamiflu, Vit C, Pan 40, MVBC,Paracetamol
U11	69	F	33	32	33	3	-	+	8.23	Diabetes mellitus	Azec, MVBC, Pan 40
[a]U12	35	F	37	33	32	11	-	-	5.85	Diabetes mellitus	DD, MUBC, Pantop 40, Tenegliptin, Metformin
[b]U13	55	M	26	24	25	6	-	+	6.47	None	Augmentin, Para, Pan40, Vit C, MVBC
[c]U14	50	M	35	28	28	6	-	-	5.47	Diabetes mellitus	NA
[a]U15	55	M	32	30	30	10	-	-	-	Hypertension, Hyperlipidemia	Azec, MVBC, Pan 40
[a]U16	65	F	32	31	32	6	-	-	8.8	None	MVBC, Pan 40
U17	66	M	-	32	-	19	-	+	7.83	Diabetes, Hypertension	Telma 40 mg, amlo 5mg, rosuvastatin 5mg, levocet10mg, MVBC, Pan 40, PCM 500mg, Teneligliptin/metformin 20/500
U18	45	M	27	26	26	11	-	+	7.08	None	PCM, Pan 40, MVBC, Azec
[a]U19	54	M	23	22	22	9	-	+	3.36	Diabetes, Immune thrombocytopenia	Azec 500, mvbc, pan 40, pcm 500
U20	45	M	30	30	30	3	-	-	-	None	NA
U21	40	M	24	22	23	5	-	+	4.07	None	Augmentin, Calpol 650, MVBC, Vit C, Pan 40
[a]U22	37	M	31	31	31	3	-	+	5.93	None	Azec, Pan 40, MVBC
U23	40	M	32	31	31	3	-	+	7.18	Diabetes	Azec, Metrogyl, Vizyla

U24	57	M	28	26	27	5	-	-	-	Diabetes, Hypertension, Stroke	Augmentin, sinarest, calpol, MVBC, Deplatt A, Zeryl, Atorva, Glimy M2, Felicita OD, Pan 40
[b]U25	27	M	20	23	19	11	-	+	7.37	None	PCM, Cough syrup, Pantaprazole
S10	46	M	-	-	32	0	-	-	-	Hypertension, Heart disease	Azec 500 mg, Pan 40, MVBC, PCM 500 mg, Vit C, Vit A
					Neg	7	+	+	4.23		
S11	58	M	-	-	21	0	-	-	3.98	Diabetes mellitus	PCM 500, Pan 40, Azee 500, MVBC
					NA	7	+	+	9.69		
S20	74	M	-	-	26	0	-	-	-	Diabetes, Hypertension	Pan, Vit C, Azec, MVBC
					Neg	7	-	-	7.2		
S21	42	M	-	-	NA	0	-	-	-	None	Azec, PCM, Pan, ondem, MVBC, Vit C, Zinc
					NA	7	+	+	6.37		
S23	47	M	-	-	28	0	-	-	4.02	Diabetes, Dyslipidemia	Azithromycin, Tamiflu, for diabetes: Rozavel, Zita plus, Glimisave
					NA	7	-	+	9.19		
S24	53	M	-	-	24	0	-	-	-	None	Azec 500, paracetamol 500, Pau 40, Ewseb 4mg, MUBC, Vit C, Zinc, cough syrup
					33	7	-	-	3.26		
S25	46	M	-	-	24	0	+	+	3.92	None	Azec, PCM, Emset, MVBC, vit C, zinc
					NA	7	+	+	8.03		
S26	18	M	-	-	33	0	-	-	-	None	NA
					Neg	7	-	+	1.74		
S27	36	M	-	-	37	0	+	+	7.79	Depression	NA
					Neg	7	-	+	7.64		
S29	33	M	-	-	25	0	-	-	3.42	None	NA
					Neg	7	-	-	5.18		
S30	49	F	-	-	NA	0	-	-	-	Hypertension, Hypothyroidism	Azee 500, Pan40, MVBC, PCM 500, Thyronorm 100ug, continue antihypertensive, Cough syrup, HCQ, Vit C, Vit A
					NA	7	-	+	6.15		
S122	71	M	-	-	32	0	-	-	NA	None	NA
						7	-	+	NA		
S123	48	M	-	-	32	0	-	-	NA	None	NA
						7	-	+	NA		
S125	66	F	-	-	28	0	-	-	NA	None	NA
						7	-	+	NA		
S132	49	M	-	-	30	0	+	+	NA	None	NA
						7	+	+	NA		
S133	64	M	-	-	24	0	-	-	NA	Diabetes,Hypertension,Asthama	NA

						7	-	+	NA		
S171	37	F	-	-	29	0	-	+	NA	None	NA
						7	+	+	NA		
S172	47	F	-	-	38	0	+	+	NA	Thyroid	NA
						7	+	+	NA		
S173	37	M	-	-	29	0	-	-	NA	None	NA
						7	+	+	NA		
S174	49	M	-	-	24	0	-	+	NA	None	NA
						7	-	+	NA		
S175	77	M	-	-	24	0	-	+	NA	Hypertension	NA
						7	+	+	NA		
S177	65	M	-	-	30	0	+	+	NA	Diabetes,Hypertension	Mezopenum 1g, Pan 40mg, MPS 500mg, Clexane 0.4cc
						7	+	+	NA		
S178	55	M	22	24	-	0	NA	NA	NA	None	NA
						7	NA	NA	NA		
S179	42	M	38	38	-	0	-	-	NA	None	NA
						7	+	+	NA		
S183	28	M	32	34	-	0	-	-	NA	None	NA
						7	-	+	NA		
S185	75	F	34	34	-	0	+	+	NA	None	NA
						7	+	+	NA		
S187	57	F	38	38	-	0	-	-	NA	None	NA
						7	+	-	NA		
S189	39	M	37	37	-	0	-	-	NA	Hypertension	NA
						7	-	-	NA		
S192	57	M	-	39	-	0	-	-	NA	None	NA
						7	-	+	NA		
S193	47	M	33	33	-	0	+	+	NA	None	NA
						7	+	+	NA		
S195	56	M	25	24	-	0	-	-	NA	Diabetes, Heart disease	NA
						7	-	+	NA		
S196	61	F	18	18	-	0	-	-	NA	Diabetes,Hypertension	NA
						7	-	-	NA		
S197	32	F	37	-	-	0	-	-	NA	None	NA
						7	-	-	NA		
S198	40	M	38	37	-	0	+	-	NA	None	NA
						7	+	-	NA		
S199	30	M	32	34	-	0	-	+	NA	None	NA
						7	-	+	NA		

C26	49	M	-	35	-	32	-	+	Plasma NA	None	NA
C28	45	M	-	34	-	35	-	+	Plasma NA	Diabetes, Hypertension, Heart disease	NA
C29	37	M	-	29	-	40	-	+	Plasma NA	None	NA
C30	27	M	-	36	-	35	-	+	2.6	None	NA
C31	37	M	-	37	-	30	-	+	7.78	COPD, Diabetes	NA
C32	20	F	-	-	-	19	-	+	4.15	NA	NA
C33	55	M	-	-	-	19	-	+	4.75	NA	NA
C42	22	M	-	-	-	33	-	-	-	NA	NA
C43	22	M	-	25	-	39	-	+	3.75	NA	NA
C44	42	M				NA	-	+	4.48	NA	NA
C45	22	M	-	24	26	37	-	+	2.63	NA	NA
C46	40	M	-	+	-	31	+	+	5.31	NA	NA
C47	20	M	-	27	-	41	-	+	4.89	NA	NA
C48	34	M				NA	-	-	-	NA	NA
C49	49	M	+	+	-	60	-	-	-	NA	NA
C50	25	F	-	37	-	62	-	+	2.92	NA	NA
C51	35	M	-	-	28	57	-	+	6.57	NA	NA
C52	24	M	-	-	-	48	+	+	-	NA	NA
C53	52	M	-	23	-	38	-	+	2.58	NA	NA
C54	24	F	-	33	-	NA	-	+	-	NA	NA
C55	34	M	NA	NA	NA	36	-	+	-	NA	NA
C56	34	M	-	30	-	40	-	+	9.47	NA	NA
C57	28	F	NA	NA	NA	52	-	+	NA	None	None
C58	33	F	NA	NA	NA	55	-	+	NA	None	None
H1	24	M	N	N	N	N	-	-	N	None	None
H2	39	M	N	N	N	N	-	-	N	None	None
H3	47	M	N	N	N	N	-	-	N	None	None
H4	45	M	N	N	N	N	-	-	N	None	None
H5	33	F	N	N	N	N	-	-	N	None	None
H6	25	F	N	N	N	N	-	-	N	None	None
H7	25	M	N	N	N	N	-	-	N	None	None
H8	29	F	N	N	N	N	-	-	N	None	None
H9	31	F	N	N	N	N	-	-	N	None	None
H10	25	F	N	N	N	N	-	-	N	Hypothyroidism	Thyronorm (88mcg)
H11	25	M	N	N	N	N	-	-	N	None	None
H12	29	M	N	N	N	N	-	-	N	None	None
H13	30	F	N	N	N	N	-	-	N	None	None

H14	25	F	N	N	N	N	-	-	N	None	None
H15	46	F	N	N	N	N	-	-	N	None	None
H16	24	M	N	N	N	N	-	-	N	None	None
H17	25	M	N	N	N	N	-	-	N	None	None
H18	26	M	N	N	N	N	-	-	N	None	None
H19	25	M	N	N	N	N	-	-	N	None	None

Footnote: U: 1st set of 25 in-patients: (U1 – U25); C: Convalescent plasma candidates (C26 – C58); S: In- patients recruited and followed up (Day 0 and Day 7, S10 – S199); NA – Not Available; N – Not Applicable; a, Asymptomatic individuals; b, Individuals transferred to other hospitals; c, Discharge information not available. All others were discharged after two consecutive negative PCR results or resolution of symptoms. Co-morbidities where documented are listed. Rapid Antibody test had 77% concordance with CLIA.

Supplemental Figure 1: Distribution of plasma TNF-α across various groups of SARS-CoV-2 infected individuals. Comparison of plasma levels of (A) in 3 healthy, seronegative controls, in-patients - IgG-/IgM- (n=22), IgG+/IgM+ (n=11), IgG+/IgM- (n=14) and sero-convalescent (n=22) groups. (B) at day 0 (n=11), day 7 (n=11), in convalescent (n=22) and in healthy (n=3) group. (C) Change in plasma levels on follow up (n=11) between day 0 and day 7. (D) Differences in plasma levels between male (n=18) and female (n=7). Red coloured symbols indicate asymptomatic individuals. Pink coloured symbols indicate IgG-/IgM- individuals in sero-convalescent group. Statistical significance was calculated by Wilcoxon matched-pairs signed rank test and unpaired Mann- Whitney U-test; *, p < 0.05; **, p < 0.01; and ***, p<0.001

A

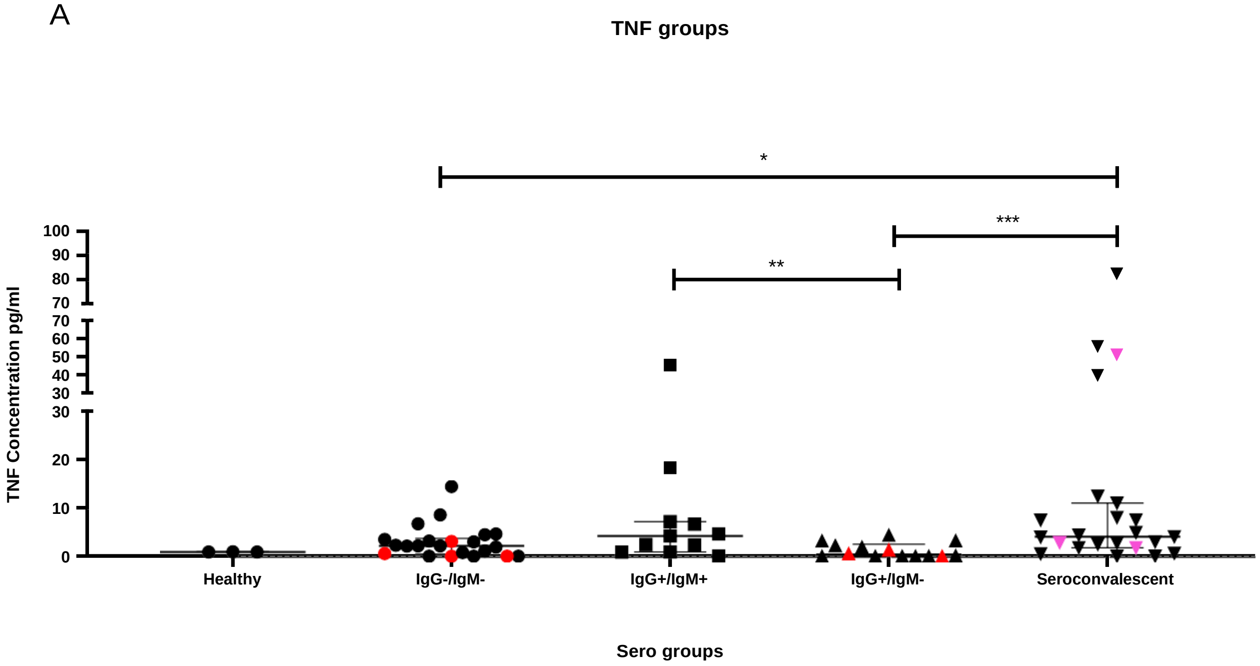

B

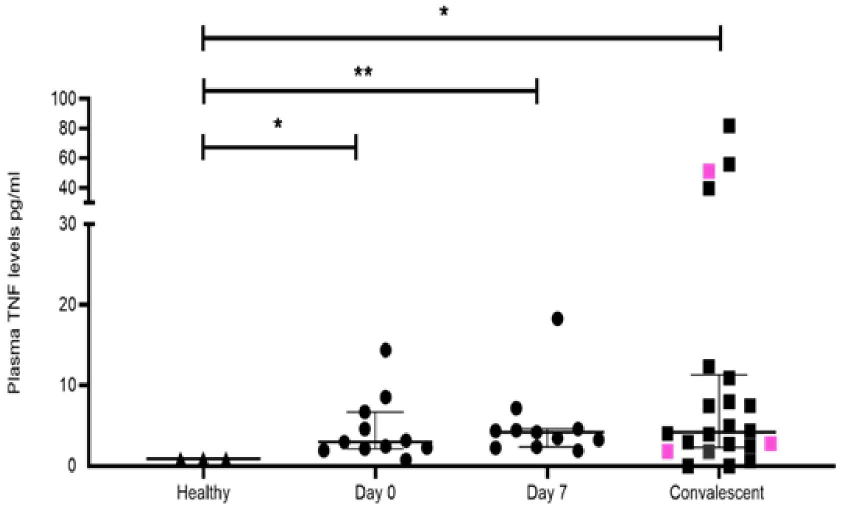

C

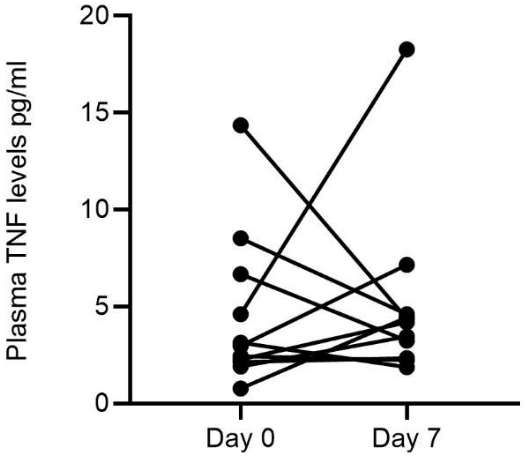

D

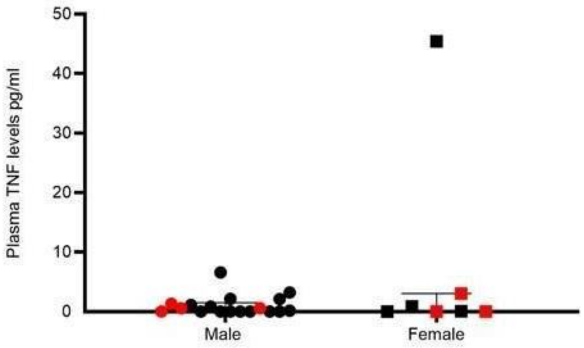

Supplemental Figure 2: Association of IL-6 with other cytokines and Ct for RT-PCR of different SARS-CoV- 2 gene. Association of plasma IL-6 levels with cytokines (n=69) (A) IL-2, (B) IL-4, (C) IL-10, (D) IFNγ, (E) TNF-α, and (F) IL-17A as well as with Ct values for viral gene targets (G) N (n=23); (H) O (n=36) and (I) S (n=50) as evaluated by real time PCR. Undetectable levels of cytokines have been assigned values of 0.

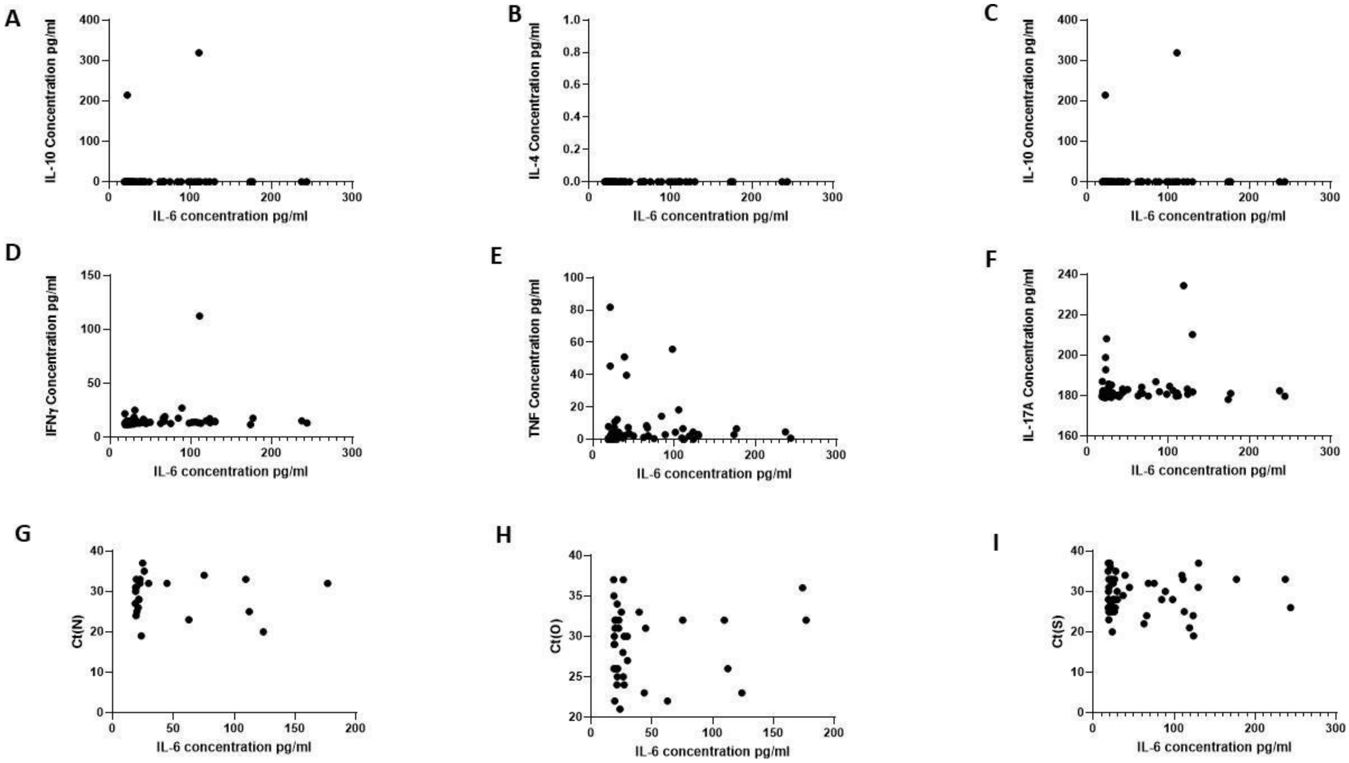

Supplemental Figure 3: Association of TNF-α with other cytokines and Ct for RT-PCR of different SARS- CoV-2 gene. Association of plasma TNF-α levels with cytokines (n=69) (A) IL-2, (B) IL-4, (C) IL-10, (D) IFNγ and (E) IL-17A as well as with Ct values for viral gene targets (F) N (n=23); (G) O (n=36) and (H) S (n=50) as evaluated by real time PCR. Undetectable levels of cytokines have been assigned values of 0.

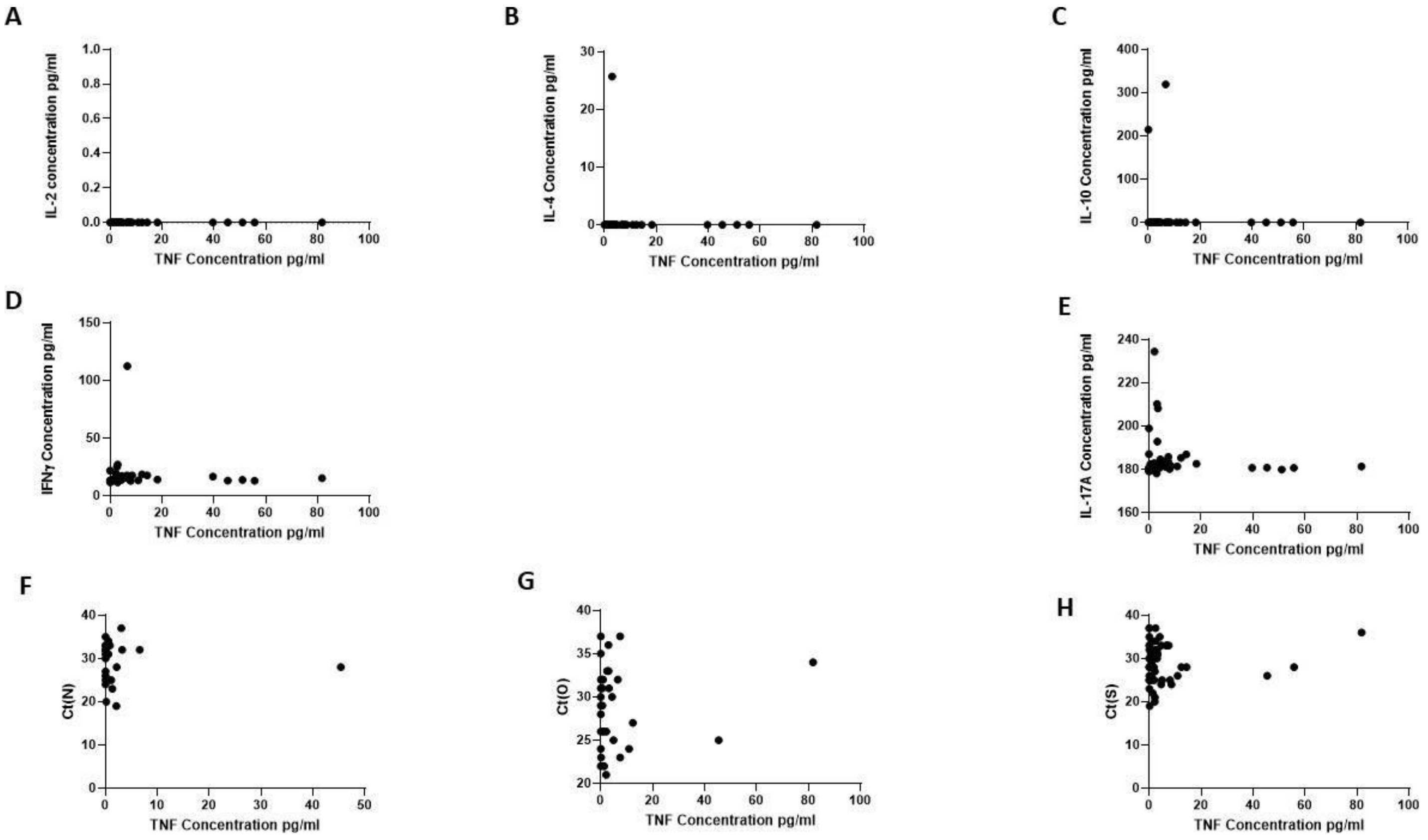

Supplemental Figure 4: Analysis of circulating lipopolysaccharides (LPS) with SARS- CoV-2 disease progression A. Comparison of LPS between IgG-/IgM- (n=10), IgG+/IgM+ (n=5), IgG+/IgM- (n=10) and convalescent (n=4) groups. B. Variation in LPS among in- patients (n=25) and in convalescent (n=4) C. Differences in LPS between male (n=21) and female (n=8). D-E. Association of circulating LPS with (D) IL-6 and (E) sMAdCAM (n=29). Red coloured symbols represent asymptomatic individuals. Statistical analysis was performed using Graphpad Prism 8.0 Mann-Whitney U-test was used to compare unpaired groups. Correlation analysis was performed using non parametric Spearman Rank Correlation test. Significance was considered at p=<0.05.

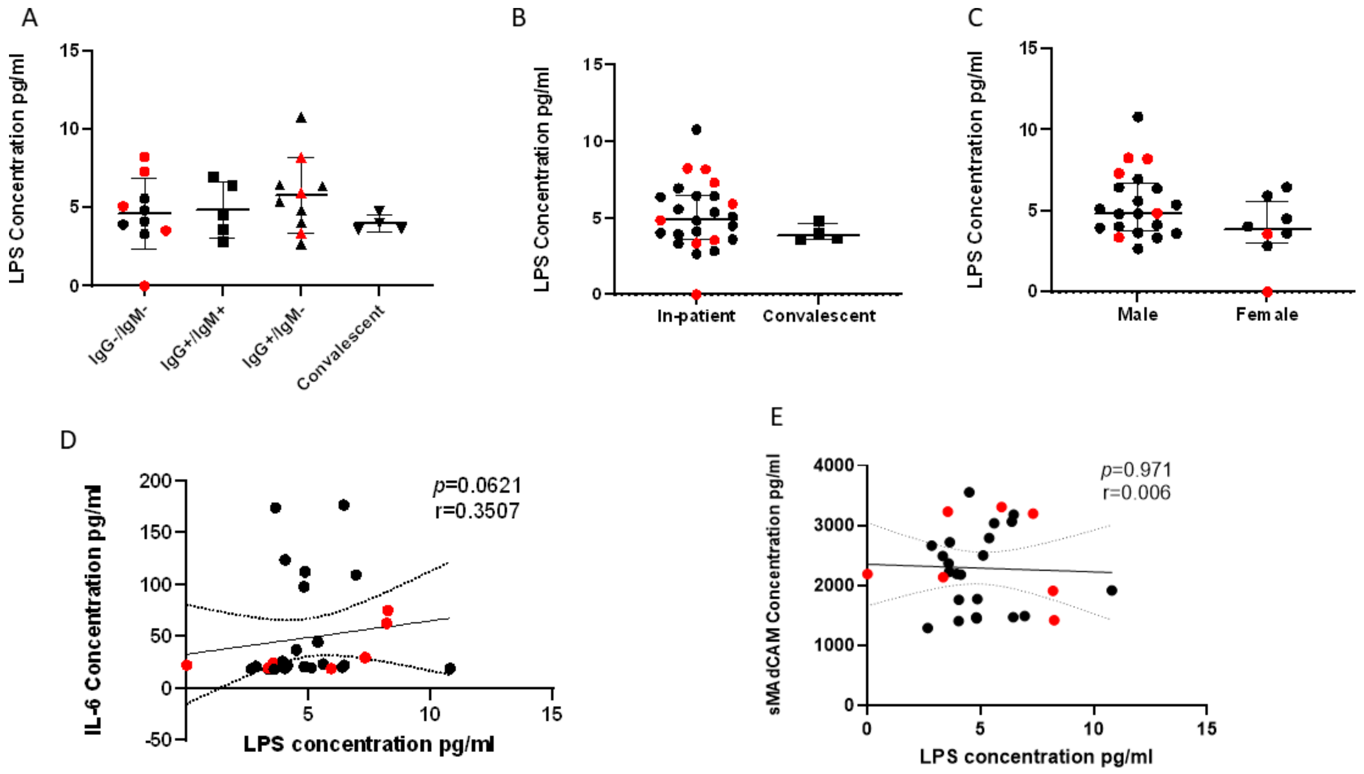

Supplement: Supplementary file 1 [file DataSheet_1.pdf]
